# Disease knowledge level is a noteworthy risk factor of anxiety and depression in patients with chronic obstructive pulmonary disease: a cross-sectional study

**DOI:** 10.1186/1471-2466-14-92

**Published:** 2014-05-28

**Authors:** Qiao Zhang, Jiangrong Liao, Xiuqing Liao, Xiuling Wu, Min Wan, Changzheng Wang, Qianli Ma

**Affiliations:** 1 Institute of Respiratory Diseases, Xinqiao Hospital, Third Military Medical University, Chongqing 400037, China; 2 Department of Respiratory Diseases, Guizhou Aerospace Hospital, Guizhou, China; 3 Department of Respiratory Diseases, Fuling Central Hospital of Chongqing, Chongqing, China; 4 Department of Respiratory Diseases, Armed Police General Hospital of Chongqing, Chongqing, China

**Keywords:** COPD, Knowledge, Anxiety, Depression, Comorbidities

## Abstract

**Background:**

Risk factors of anxiety and depression symptoms in patients with chronic obstructive pulmonary disease (COPD) have been widely researched, but most of them cannot be addressed clinically. The aim of this study was to investigate whether COPD knowledge level is a risk factor of anxiety and/or depression in COPD patients in addition to functional capacity and quality of life, and to determine the key topics of COPD knowledge.

**Methods:**

A total of 364 COPD patients from four centers were recruited into this cross-sectional survey. Subjects’ general medical information, assessments of lung function, dyspnea, quality of life, and exercise capacity, and responses to the Hospital Anxiety and Depression Scale (HAD) and the Bristol COPD Knowledge Questionnaire (BCKQ) were collected. Partial correlation analysis was performed, and a multivariable model testing risk factors of anxiety and depression as well as a multivariable model of 13 topics of knowledge derived from BCKQ were constructed.

**Results:**

Subjects with anxiety or depression were more likely to have less COPD knowledge. Partial correlation analysis revealed that HAD score was negatively correlated with BCKQ score (rho = −0.153, *P* = 0.004). BCKQ score was significant in the multivariable model that tested risk factors of anxiety and depression (*P* = 0.001, OR = 0.944). Topics of epidemiology (*P* < 0.001, OR = 0.653) and infections (*P* = 0.006, OR = 0.721) were significant in the multivariable model evaluating 13 topics.

**Conclusions:**

The level of patients’ disease knowledge is a significant risk factor of anxiety and depression in COPD patients. Epidemiology and infections are key topics of COPD knowledge to target in the Chinese population.

**Trial registration:**

ChiCTR-OCS-12002518

## Background

Anxiety and depression are common psychological disturbances affecting a substantial number of patients with COPD, with reported prevalence rates of 10–57% for anxiety and 10–59% for depression [1-4]. Both are significantly associated with increased physical disability and morbidity, decreased health status, and decreased compliance with medical treatment [4-8].

Successful psychological or psychiatric interventions could address anxiety and depression. Exploring the risk factors of COPD patients with anxiety and/or depression may be valuable for understanding disease processes and improving disease prevention.

Some risk factors of anxiety and depression symptoms in COPD patients have been widely researched, including younger age, female gender, current smoker status, bronchitic phenotype, more advanced disease, cardiovascular comorbidities, and worse functional capacity and quality of life [1,2,9,10]. However, the overall explanatory value of these risk factors is weak. New risk factors that might have previously been ignored should be investigated. The finding that COPD patients’ anxiety and depression symptoms improved following pulmonary rehabilitation provides a valuable clue [11-13].

Patient education, an essential element of pulmonary rehabilitation, relates to improving COPD patients’ disease knowledge level [14]. The relief of anxiety and depression symptoms may be associated with patient education affecting COPD patients’ disease knowledge level. We have found that COPD patients with greater disease knowledge had fewer symptoms of anxiety and depression in our COPD disease management plan (data not shown). We hypothesize that COPD patients’ disease knowledge (including knowledge of epidemiology, aetiology, symptoms, treatment, and disease management of COPD) is another risk factor related to anxiety and depression symptoms.

In this cross-sectional study, we investigate whether COPD knowledge level is a risk factor of anxiety and depression symptoms in COPD patients alongside functional capacity and quality of life. We examined the effect of 13 key topics of COPD knowledge measured by BCKQ, including (1) epidemiology, (2) aetiology, (3) symptom, (4) breathlessness, (5) phlegm, (6) infections, (7) exercise, (8) smoking, (9) vaccination, (10) inhaled bronchodilators, (11) antibiotics, (12) oral steroids, and (13) inhaled steroids [15].

## Methods

### Study design

This study was a cross-sectional survey. It was conducted at four centers in two districts, in accordance with the Declaration of Helsinki and good clinical practice guidelines, and was approved by the medical ethics committee of the second affiliated hospital of Third Military Medical University. All patients were enrolled from an outpatient clinic and gave informed consent. The study was registered in the Chinese Clinical Trial Registry (http://www.chictr.org, Registration no: ChiCTR-OCS-12002518).

### Participants and measurements

All patients with COPD were consecutively recruited from four outpatient clinics in Guizhou Province, Chongqing Municipality, China from September 2012 to June 2013. All participants were diagnosed with COPD in accordance with the Global Initiative for Chronic Obstructive Lung Disease strategy document [16] and confirmed by post-bronchodilator FEV1/FVC of less than 0.7. Patients with self-reported comorbidities or COPD exacerbation in the previous 4 weeks were excluded.

Subjects’ general medical information was collected, including age, gender, duration of disease, smoking history, and highest educational qualification. Lung function tests were performed according to American Thoracic Society/European Respiratory Society (ATS/ERS) guidelines [17]. In addition, patient-reported outcomes were measured, including assessment of dyspnea (modified Medical Research Council Dyspnea scale (mMRC)) [18], psychological disturbance (HAD scale) [19], quality of life (COPD Assessment Test (CAT)) [20], and BCKQ [15]. All questionnaires were completed by subjects independently with adequate space, a firm writing surface, and a pencil. If the patient was illiterate or had other complications that prevented them from completing the questionnaire, the investigator obtained patient responses by reading each question out loud verbatim, followed by the corresponding response categories, and entering the patient’s responses. The investigator did not influence patient responses. Exercise capacity was assessed using the six-minute walking distance test (6MWD) carried out according to ATS guidelines [21]. Subjects were ordered to walk as rapidly as possible along a solid and flat corridor for 6 minutes.

### Measurement of anxiety and depression

The HAD scale was used to assess anxiety and depression symptoms in all eligible subjects [19]. It has been used extensively to screen psychiatric morbidity and has high validity when used as a screening instrument in outpatients [22]. HAD consists of two 7-item subscales: 7 questions relate to anxiety (HAD-A) and 7 to depression (HAD-D). Scores range from 0 to 21 for each subscale, and a score of 8 or higher on either subscale indicates possible pathology. Using this cutoff, HAD shows high sensitivity and specificity in Chinese patients [23].

### Measurement of COPD patients’ disease knowledge level

BCKQ was used to assess eligible COPD patients’ disease knowledge level. It consists of 13 subscales, each of which assesses a topic of COPD knowledge: (1) epidemiology, (2) aetiology, (3) symptom, (4) breathlessness, (5) phlegm, (6) infections, (7) exercise, (8) smoking, (9) vaccination, (10) inhaled bronchodilators, (11) antibiotics, (12) oral steroids, and (13) inhaled steroids. Each topic contains five questions. Scores range from 0 to 65, with higher scores suggesting higher disease knowledge level in COPD patients. BCKQ tests knowledge appropriate for COPD patients and can be used as a broad cross-sectional survey instrument [15]. Prior to this study, we translated, retranslated, and piloted the questionnaire in a Chinese population [24].

### Statistical analysis

Means and standard deviations were computed for variables including age, pack-year history, duration of COPD, CAT, mMRC, BCKQ, FEV1, FEV1%, postFEV1, postFEV1%, and 6MWT. An independent samples t-test was used to compare continuous variables between groups (with or without anxiety/depression). A chi-square test was used to test categorical variables. Spearman’s correlation coefficients were calculated between HAD total score and descriptor variables. Partial correlation analysis was performed to assess the correlation between HAD and target descriptor variables while controlling for the effect of other variables. [Additional file 1 provides the details of this partial correlation analysis]. A combined analysis of patient-reported outcomes, clinically based descriptors, and lung function test results was performed using a stepwise logistic regression model. The *P*-values necessary for entry into the model and to remain in the model were both *P* < 0.1. The same method was used to assess the 13 topics of the BCKQ.

## Results

### Subject demographics

A total of 416 subjects were eligible for the study. Fifty two subjects refused to be included. A total of 364 subjects provided informed consent and entered the study. Five of these subjects were excluded due to inadequate completion or loss of data. Therefore, 359 participants were analyzed. Thirty two participants were female. The mean age was 65.64 ± 7.60 years. A total of 95 patients (26.46%) had an HAD-A and/or HAD-D score of ≥ 8. The mean CAT score was 16.42 ± 7.20, the mean mMRC score was 1.53 ± 0.85, and the mean BCKQ score was 30.36 ± 5.59 - only 46% of the full mark possible. In total, 15.32% had educational qualifications at junior college level or above. No subjects were receiving treatment for their anxiety/depression at the time of assessment. More demographic details are listed in Table 1. Subjects with anxiety/depression were more likely to be younger, female, to have worse quality of life, increased dyspnea, and a lower level of disease knowledge about COPD (see below).

**Table 1:** Characteristics of subjects stratified by presence of anxiety and/or depression status

	**Total**	**Anxiety and/or Depression status**		***P *value**
		**Yes (n = 95)**	**No (n = 264)**	
Age, mean ± SD	65.64 ± 7.60	64.25 ± 7.84	66.14 ± 7.47	0.038
Gender, n (male/female)	327/32	77/18	250/14	<0.001
Pack-year history, mean ± SD	33.65 ± 18.37	32.46 ± 20.28	34.08 ± 17.66	0.463
Duration of COPD, years, mean ± SD	6.69 ± 5.32	6.77 ± 6.19	6.66 ± 4.98	0.859
CAT, mean ± SD	16.42 ± 7.20	20.17 ± 6.50	15.06 ± 6.97	<0.001
mMRC, mean ± SD	1.53 ± 0.85	1.86 ± 0.86	1.42 ± 0.82	<0.001
BCKQ, mean ± SD	30.36 ± 9.59	26.85 ± 9.16	31.63 ± 9.45	<0.001
HAD-A, mean ± SD	3.54 ± 3.74	7.87 ± 3.69	1.98 ± 2.24	<0.001
HAD-D, mean ± SD	4.17 ± 3.76	8.97 ± 2.69	2.45 ± 2.31	<0.001
FEV1, L, mean ± SD	1.11 ± 0.44	1.04 ± 0.41	1.14 ± 0.45	0.062
FEV1% predict, mean ± SD	42.88 ± 25.56	39.12 ± 14.77	44.23 ± 28.36	0.094
Post FEV1, L, mean ± SD	1.22 ± 0.47	1.16 ± 0.47	1.24 ± 0.46	0.133
Post FEV1% predict, mean ± SD	46.77 ± 25.88	43.37 ± 116.19	48.00 ± 28.50	0.135
6MWD, m, mean ± SD	429.64 ± 115.39	431.38 ± 124.34	429.01 ± 112.24	0.864
Primary school, n (%)	106 (29.53%)	27 (28.42%)	79 (29.92%)	0.783
Junior high school, n (%)	129 (35.93%)	42 (44.21%)	87 (32.95%)	0.050
Senior high school, n (%)	59 (16.43%)	14 (14.74%)	45 (31.78%)	0.603
Junior college or above, n (%)	55 (15.32%)	8 (8.42%)	47 (17.80%)	0.029
Other, n (%)	10 (2.79%)	4 (4.21%)	6 (2.27%)	0.301

*Abbreviations*: *CAT* COPD Assessment Test, *mMRC* modified Medical Research Council, *BCKQ* Bristol COPD Knowledge Questionnaire, *HAD* Hospital Anxiety and Depressive Scale, *HAD-A* HAD-anxiety, *HAD-D* HAD-Depression, *FEV1* Forced Expiratory Volume in 1 Second, *postFEV1* post-bronchodilator FEV1, *6MWD* Six-Minute Walking Distance Test; Definition of Anxiety and/or Depression Status: HAS-A ≥8 and/or HAD-D score ≥8.

### Correlation of HAD total score with characteristics of subjects

Spearman correlation analysis revealed significant correlations between HAD score and age, gender, CAT score, mMRC score, BCKQ score, FEV1, FEV1% predicted, post-bronchodilator FEV1, post-bronchodilator FEV1% predicted, and 6MWD.

A partial correlation analysis revealed that higher HAD total score correlated with female gender (rho = 0.376, *P* < 0.001), worse quality of life (CAT score, rho = 0.351, *P* < 0.001), increased dyspnea (mMRC, rho = 0.109, *P* = 0.042), and lower level of COPD knowledge (BCKQ, rho = −0.153, *P* = 0.004). No other variables significantly correlated with HAD scores, including age, smoke history, duration of COPD, FEV1, FEV1% predicted, post-bronchodilator FEV1, post-bronchodilator FEV1% predicted, and 6MWD (Table 2).

**Table 2:** Correlation between HAD total score and subject characteristics

	**Spearman’s rho**	***P *value**	**Partial correlations**	***P *value**
Age	−0.122	0.021	−0.036	0.509
Gender^†^	0.295	0.000	0.376	<0.001
Pack-year history	−0.029	0.586	0.051	0.344
Duration of COPD	0.014	0.799	−0.043	0.419
CAT	0.495	0.000	0.351	<0.001
mMRC	0.309	0.000	0.109	0.042
BCKQ	−0.217	0.000	−0.153	0.004
FEV1 (L)	−0.157	0.003	−0.063	0.243
FEV1% predict	−0.174	0.001	0.043	0.425
PostFEV1 (L)	−0.145	0.006	0.070	0.194
PostFEV1% predict	−0.158	0.003	−0.051	0.341
6MWT	−0.112	0.034	0.014	0.789

*Abbreviations*: *CAT* COPD Assessment Test, *mMRC* modified Medical Research Council, *BCKQ* Bristol COPD Knowledge Questionnaire, *HAD* Hospital Anxiety and Depressive Scale, *FEV1* Forced Expiratory Volume in 1 Second, *postFEV1* post-bronchodilator FEV1, *6MWD* Six-Minute Walking Distance Test.

^†^Set “male” = 0, “female” = 1.

### Risk factors of anxiety and depression

A multivariable model testing risk factors of anxiety and depression explored factors including age, gender, CAT, mMRC, BCKQ score, FEV1, FEV1% predicted, post-bronchodilator FEV1, post-bronchodilator FEV1% predicted, and 6MWD. Gender (“male” = 0, “female” = 1, *P* = 0.001, OR = 3.781), CAT score (*P* < 0.001, OR = 1.087), mMRC score (*P* = 0.050, OR = 1.386) and BCKQ score (*P* = 0.001, OR = 0.944) were significant in this model. In accordance with our hypotheses, level of COPD knowledge was a risk factor of anxiety and/or depression symptoms in COPD patients alongside functional capacity and quality of life. However, the overall predictive capability of the model was very weak (Hosmer and Lemeshow Test, *P* = 0.128).

### Topics of COPD knowledge and subjects’ anxiety or depression

The 13 topics of COPD knowledge measured by BCKQ were assessed. Subjects with anxiety and/or depression had lower scores in topics (1) epidemiology, (3) symptom, (4) breathlessness, (5) phlegm, (6) infections, (9) vaccination, (10) inhaled bronchodilators, and (11) antibiotics. These topics were used to construct the multivariable model. (1) Epidemiology (*P* < 0.001, OR = 0.653) and (6) Infections (*P* = 0.006, OR = 0.721) were significant in this model. The *P* value of Hosmer and Lemeshow Test of this model was 0.919 (Table 3).

**Table 3:** Topic score in BCKQ for subjects stratified by presence of anxiety and/or depression status

**Topic**	**Total**	**Anxiety and/or depression status**		***P *value**
		**Yes (n = 95)**	**No (n = 264)**	
1 Epidemiology^†^	2.91 ± 1.06	2.39 ± 1.07	3.10 ± 1.02	<0.001
2 Aetiology	3.56 ± 1.04	3.37 ± 1.07	3.63 ± 1.03	0.098
3 Symptom	2.79 ± 1.01	2.42 ± 0.88	2.92 ± 1.04	0.001
4 Breathlessness	2.92 ± 0.91	2.72 ± 0.96	2.99 ± 0.88	0.047
5 Phlegm	3.23 ± 1.32	2.78 ± 1.42	3.39 ± 1.26	0.003
6 Infections^‡^	2.28 ± 1.11	1.83 ± 1.10	2.44 ± 1.09	< 0.006
7 Exercise	2.96 ± 1.22	2.78 ± 1.20	3.03 ± 1.23	0.173
8 Smoking	2.97 ± 0.95	2.80 ± 0.99	3.04 ± 0.93	0.102
9 Vaccination	1.75 ± 1.15	1.49 ± 1.07	1.85 ± 1.16	0.036
10 Inhaled bronchodilators	1.33 ± 1.06	1.03 ± 1.05	1.44 ± 1.05	0.009
11 Antibiotics	1.48 ± 1.11	1.14 ± 0.97	1.61 ± 1.14	0.003
12 Oral steroids	1.27 ± 1.06	1.28 ± 1.12	1.27 ± 1.04	0.963
13 Inhaled steroids	0.90 ± 0.89	0.83 ± 0.88	0.92 ± 0.90	0.480

Definition of Anxiety and/or Depression Status: HAS-A ≥ 8 and/or HAD-D score ≥ 8.

^**†**^LogisticRegression:*P* < 0.001, OR = 0.653.

^**‡**^LogisticRegression: *P* = 0.006, OR = 0.721.

## Discussion

In this study, 26.46% of COPD patients had anxiety and/or depression. Subjects with anxiety and/or depression were significantly more likely to be younger, female, and to have worse quality of life and increased dyspnea. This is consistent with previous studies. Worse anxiety and/or depression symptoms (with higher HAD total scores) significantly correlated with younger age, female patients, worse quality of life, increased dyspnea, increased severity of airway limitation, and lower 6MWD [2,9,10].

The key finding of this study is that the level of patients’ COPD knowledge was another risk factor of anxiety and/or depression alongside functional capacity, quality of life, and gender. A higher HAD score correlated with lower COPD knowledge (BCKQ, rho = −0.153, *P* = 0.004). Previous studies mainly focused on the relationship between anxiety and/or depression and COPD patients’ clinical features, including patient-reported outcomes, demographic factors, and objective measures [2,9,10,25]. Most risk factors identified previously could not be addressed. The ECLIPSE study concluded “subjective measures, represented by quality of life and symptom, were stronger determinants of depression than objective measures, such as lung function and biologic and physiologic markers” [10]. Level of COPD knowledge, a subjective measure, appears to be a new risk factor that was previously ignored and that can be addressed.

However, it is important to point out that the overall explanatory value of our final four-variable multivariable regression model was weak. Therefore, although we have confirmed that level of COPD knowledge was a new risk factor, none of the factors we measured strongly explained the presence of anxiety and/or depression in patients with COPD. Based on these results, we hypothesize that in COPD patients, anxiety and depression are primarily driven by the patient’s perception of serious chronic disease and the mindset of facing this troubled state. Further research should be conducted to increase knowledge in this area.

There is controversy over whether the education interventions in clinical practice were efficacious. For example, in a randomized trial of exercise, stress management, and education, investigators did not find the benefit of reducing anxiety in the education group [26], while another randomized trial of cognitive behavioral therapy and COPD education reported that both forms of education were efficacious in reducing anxiety or depressive symptoms. A limited number of trials have been performed [27], all trials used different methods to educate patients, and none assessed actual change in COPD knowledge. These defects may have led to different results and points of view. The content and frequency of education as well as educational methods all require further study.

To evaluate key topics of COPD knowledge to address in educational interventions, a further logistic model based on the 13 topics of BCKQ was constructed. Although subjects with anxiety and/or depression had lower scores in (1) epidemiology, (3) symptom, (4) breathlessness, (5) phlegm, (6) infections, (9) vaccination, (10) inhaled bronchodilators, and (11) antibiotics, knowledge of topics (1) epidemiology and (6) infections was found to be a risk factor for anxiety and/or depression in COPD patients.

The epidemiology topic contains questions such as: “In COPD, the word ‘chronic’ means that it is severe”. However, the reason that the epidemiology category was significant is unknown, we conjecture that this misunderstanding of the disease may be involved. A patient who has misunderstood the meaning of the name COPD may make incorrect interpretations, such as “I am going to die”, “I am past all hope”, and “All these treatments are useless to me”, when he or she is experiencing dyspnea. This in turn may lead to a heightened state of physiological arousal accompanied by further sensations and misinterpretations.

The category ‘infections’ addresses the relationship between infections and treatment of COPD exacerbation. The causes of the significance of this factor were also unclear. We conjecture that this may be a special topic in China. Anti-inflammation and anti-infection have the same meaning for some Chinese patients. They consequently regard COPD as an infectious disease. Because most Chinese patients have easy access to antibiotics, this may result in antibiotics abuse and poor outcomes. COPD patients who understand infection in this way may have more negative thoughts and emotions than do patients who can interpret, understand, and treat COPD correctly. This suggests that patients from different cultural backgrounds may have different results.

We acknowledge the limitations in our study. First, the overall level of COPD knowledge and education was low, which accurately reflects the current situation of COPD patients in West China [24]. In addition, gender was imbalanced. The number of male patients was nearly 10 times higher than the number of female patients. This may result from the gender ratio characteristic of COPD. There was a 3:1 ratio of men to women in the epidemiological investigation, and this is comparable with that of another multicenter study held in China [28]. The influence of gender imbalance was limited to the relationship between the level of COPD knowledge and anxiety/depression.

## Conclusions

A combination of anxiety and depression symptoms is common in COPD patients. In this study, we found that the level of COPD patients’ disease knowledge was a risk factor of anxiety and/or depression alongside functional capacity and quality of life. Key topics of COPD knowledge that could be addressed were (1) epidemiology and (6) infections. Relevant topics may differ in patients with different cultural backgrounds. Our study indicates that the relationship between COPD knowledge level, mental health, and education strategy is valuable to investigate in the future.

## Abbreviations

COPD: Chronic obstructive pulmonary disease; BCKQ: Bristol COPD knowledge questionnaire; HAD: Hospital anxiety and depression; HAD-A: HAD-Anxiety; HAD-D: HAD-Depression; CAT: COPD assessment test; mMRC: Modified Medical Research Council Dyspnea Scale; 6MWD: Six-minute walking distance test; FEV1: Forced expiratory volume in 1 second; postFEV1: Post-bronchodilator FEV1; FVC: Forced vital capacity.

## Competing interests

The authors declare that they have no conflicts of interest in relation to this article. J.R. Liao, X.L. Wu, X.Q. Liao, and M. Wan declare that they have no competing interests. Q. Zhang has received lecture fees and travel grants from AstraZeneca and GlaxoSmithKline (GSK). C.Z. Wang and Q.L. Ma have received lecture fees and travel grants from AstraZeneca, Boehringer Ingelheim, GSK, and Novartis, and they have received lecture fees from Takeda, Aerocrine, Daiichi Sankyo, and Merck Sharp & Dohme (MSD).

## Authors’ contributions

QZ drafted the manuscript, participated in patient recruitment, collection of subjects’ general medical information, HAD scale, BCKQ scale, and performed the partial correlation analysis. MW constructed multivariable models testing risk factors of anxiety/depression and the 13 topics from BCKQ. QM conceived of the study, performed the statistical analysis, participated in the design and coordination of the study, and helped to draft the manuscript. CW, JL, XL, and XW participated in the design of the study, participated in patient recruitment and collecting subjects’ general medical information, HAD scale, and BCKQ scale. All authors read and approved the final manuscript.

## Pre-publication history

The pre-publication history for this paper can be accessed here:

http://www.biomedcentral.com/1471-2466/14/92/prepub

## Supplementary Material

Additional file 1 **Details of the variables in partial correlations analysis.** Description of data: All details of the variables in partial correlation analysis were described. Click here for file
