# Supplementary material for: Disease knowledge level is a noteworthy risk factor of anxiety and depression in patients with chronic obstructive pulmonary disease: a cross-sectional study
Source: BMC Pulm Med. 2014 May 28;14:92. doi: 10.1186/1471-2466-14-92 (PMC4041907; doi:10.1186/1471-2466-14-92)
Supplement: Additional file 1 — Details of the variables in partial correlations analysis. Description of data: All details of the variables in partial correlation analysis were described. [file 1471-2466-14-92-S1.docx]

Details of the variables in partial correlations analysis

| No. | Variable A | Variable B | Control |
| --- | --- | --- | --- |
| 1 | HAD total score | Age | Gender, Pack-year history, Duration of COPD, CAT, mMRC, BCKQ, FEV1, FEV1%predict, postFEV1, postFEV1%predict and 6MWT |
| 2 |  | Gender | Age, Pack-year history, Duration of COPD, CAT, mMRC, BCKQ, FEV1, FEV1%predict, postFEV1, postFEV1%predict and 6MWT |
| 3 |  | Pack-year history | Age, Gender, Duration of COPD, CAT, mMRC, BCKQ, FEV1, FEV1%predict, postFEV1, postFEV1%predict and 6MWT |
| 4 |  | Duration of COPD | Age, Gender, Pack-year history, CAT, mMRC, BCKQ, FEV1, FEV1%predict, postFEV1, postFEV1%predict and 6MWT |
| 5 |  | CAT | Age, Gender, Pack-year history, Duration of COPD, mMRC, BCKQ, FEV1, FEV1%predict, postFEV1, postFEV1%predict and 6MWT |
| 6 |  | mMRC | Age, Gender, Pack-year history, Duration of COPD, CAT, BCKQ, FEV1, FEV1%predict, postFEV1, postFEV1%predict and 6MWT |
| 7 |  | BCKQ | Age, Gender, Pack-year history, Duration of COPD, CAT, mMRC, FEV1, FEV1%predict, postFEV1, postFEV1%predict and 6MWT |
| 8 |  | FEV1 | Age, Gender, Pack-year history, Duration of COPD, CAT, mMRC, BCKQ, FEV1%predict, postFEV1, postFEV1%predict and 6MWT |
| 9 |  | FEV1%predict | Age, Gender, Pack-year history, Duration of COPD, CAT, mMRC, BCKQ, FEV1, postFEV1, postFEV1%predict and 6MWT |
| 10 |  | postFEV1 | Age, Gender, Pack-year history, Duration of COPD, CAT, mMRC, BCKQ, FEV1, FEV1%predict, postFEV1%predict and 6MWT |
| 11 |  | postFEV1%predict | Age, Gender, Pack-year history, Duration of COPD, CAT, mMRC, BCKQ, FEV1, FEV1%predict, postFEV1 and 6MWT |
| 12 |  | 6MWT | Age, Gender, Pack-year history, Duration of COPD, CAT, mMRC, BCKQ , FEV1, FEV1%predict, postFEV1 and postFEV1%predict |
